# Models of *ex vivo* explant cultures: applications in bone research

**DOI:** 10.1038/bonekey.2016.49

**Published:** 2016-06-29

**Authors:** Silvia Marino, Katherine Ann Staines, Genevieve Brown, Rachel Anne Howard-Jones, Magdalena Adamczyk

**Affiliations:** 1 Academic Unit of Bone Biology, Department of Oncology and Metabolism, Mellanby Centre for Bone Research, Medical School, The University of Sheffield, Sheffield, UK; 2 Roslin Institute and R(D)SVS, The University of Edinburgh, Edinburgh, UK; 3 Department of Biomedical Engineering, Columbia University, New York, USA; 4 Oral and Biomedical Sciences, College of Biomedical and Life Sciences, Cardiff University, Cardiff, UK

## Abstract

*Ex vivo* explant culture models are powerful tools in bone research. They allow investigation of bone and cartilage responses to specific stimuli in a controlled manner that closely mimics the *in vivo* processes. Because of limitations in obtaining healthy human bone samples the explant growth of animal tissue serves as a platform to study the complex physico-chemical properties of the bone. Moreover, these models enable preserving important cell–cell and cell–matrix interactions in order to better understand the behaviour of cells in their natural three-dimensional environment. Thus, the use of bone *ex vivo* explant cultures can frequently be of more physiological relevance than the use of two-dimensional primary cells grown *in vitro*. Here, we describe isolation and *ex vivo* growth of different animal bone explant models including metatarsals, femoral heads, calvaria, mandibular slices and trabecular cores. We also describe how these explants are utilised to study bone development, cartilage and bone metabolism, cancer-induced bone diseases, stem cell-driven bone repair and mechanoadaptation. These techniques can be directly used to understand mechanisms linked with bone physiology or bone-associated diseases.

## Introduction

**Figure 1:**
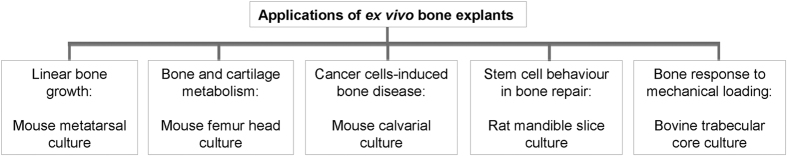
Models of animal *ex vivo* bone explant cultures and their applications.

The bone is a complex and specialised connective tissue in a state of dynamic equilibrium that, together with cartilage, forms the skeletal system. Active reorganisation of bone microstructure is achieved through bone remodelling or modelling, processes that are mediated by osteoclasts, osteoblasts and osteocytes.^1^ Remodelling represents coordinated changes in bone resorption and formation, which occur throughout life, in order to maintain health and integrity of the skeleton. Bone modelling is an adaptation of bone to external stimuli and is generally limited to growth and responses to mechanical loading. *Ex vivo* growth of bone explants offers a unique opportunity to study these mechanisms in a controlled experimental setting where both the three-dimensional organisation as well as the cellular diversity of the bone are preserved. In addition, the *ex vivo* cultures can help the investigation of mechanisms underlying bone growth, bone and cartilage matrix turnover, mechanical loading, or interactions with other cell types and advance our understanding of bone physiology. The aim of this collective protocol is to provide a detailed description of the procedures that are currently used to isolate, culture and characterise a variety of animal bone explant culture models and include the applications of these models in bone research (Figure 1). This protocol combines previously published protocols and those optimised in our laboratories and offers the technical hints and suggestions for each of the methods. This protocol also provides a list of additional reading that should help the researcher with establishing these procedures at their home institution.

### *Ex vivo* explant model to study linear bone growth

The mouse metatarsal culture model can be used to improve our understanding of bone development. This model, pioneered by Burger and Van-Delft in the Netherlands (1976),^2^ is a highly physiological *ex vivo* model for studying endochondral ossification, the process by which the cartilage scaffold is replaced by mineralised bone. This process is tightly regulated in healthy individuals in order to prevent abnormal development and/or longitudinal bone growth.^3^ The metatarsal model allows for the study of linear bone growth, as the growth rate of the bones in culture mimics that seen *in vivo*. Moreover, this model enables investigation of chondrocyte proliferation and hypertrophy, extracellular matrix production and tissue mineralisation. The method described in this protocol relates to murine metatarsal explant cultures; however, there are numerous papers detailing similar cultures in rats^4^ (Method 1).

### *Ex vivo* explant model to study bone and cartilage metabolism

The mouse femoral head culture model can be used to investigate the expression and turnover of specific markers within bone and cartilage. This is useful to help understand the mechanisms linked with pathogenesis of osteoarthritis, where there is a clear association between articular cartilage degeneration and subchondral bone changes.^5^,^6^ This model was developed by Stanescu and Leibovich (1982),^7^ and over the years it has been primarily used to understand mechanisms of cartilage breakdown. For instance, this model was effectively applied by Glasson *et al*.^8^ to demonstrate the importance of aggrecan proteolytical cleavage and neoepitope formation during osteoarthritis progression.^8^ Uniquely, the mouse femoral head culture model can also be used for simultaneous investigation of bone modelling and cartilage extracellular matrix degradation occurring during explant culture^9^ (Method 2).

### *Ex vivo* explant model to study cancer cell-induced bone disease

The mouse calvaria *ex vivo* culture model has been routinely used to investigate bone resorption,^10^ bone formation^11^ and bone regeneration/healing process.^12^ By using an adaptation of the calvaria explant culture model, it is also possible to study cancer-induced bone metastases *ex vivo* as shown by Sophocleous *et al*.^13^ and others. Cancer to bone metastasis is mediated by the secretion of soluble factors by cancer cells, which stimulate the proliferation and activity of osteoblasts and osteoclasts, affecting both bone formation as well as bone resorption. The method described in this protocol gives an example of the isolation and culture of calvaria derived from mouse pups; however, similar cultures can be established using the calvarial bones obtained from rats^14^ or chicken embryos^15^ (Method 3).

### *Ex vivo* explant model to study stem cell behaviour during bone repair

A rat mandible slice model was initially established to investigate inflammatory bone destruction.^16^ This model was adapted by Colombo and colleagues to enable the study of dental pulp stem cell (DPSC) behaviour in an *ex vivo* environment.^17^ DPSCs have characteristics similar to mesenchymal stem cells^18^,^19^ and display migratory and odontoblast differentiation capacity in response to tissue damage.^19^,^20^,^21^ Studying DPSC behaviour in an *ex vivo* mandible slice enables understanding of their multi-potency and how they may initiate bone repair in response to injury (Method 4).

### *Ex vivo* explant model to study bone response to loading

The bovine trabecular cores culture model is extremely useful when investigating bone changes in response to mechanical stimulation. Osteocytes – the mechanosensors in bone—direct these processes by regulating osteoclast and osteoblast activities;^22^ yet, few models isolate osteocyte interactions. The model described herein allows for the study of osteocyte–osteoblast interactions in a long-term *ex vivo* culture and will further our understanding of bone mechanoadaptation.^23^,^24^ Although the method described here utilises perfusion of bovine bone cores,^25^,^26^,^27^,^28^ similar systems using rabbit trabecular bone explants and whole bone organ cultures have recently been reported^29^,^30^ (Method 5).

## Materials and methods

### Dissections

All dissections should be conducted aseptically using sterile equipment and in a dissection hood or tissue culture hood to maintain a sterile environment.

### Cell and explant cultures

All tissue culture procedures are carried out in a sterile laminar flow tissue culture hood. Cells and *ex vivo* cultures are maintained in an incubator at 37 °C and 5% CO_2_. Cell culture reagents should be sterile and pre-warmed to 37 °C unless otherwise indicated in the protocols. Cells can be grown in the standard tissue culture-treated plastic culture dishes/plates unless otherwise indicated.

### Use of animals

All procedures described in this protocol were performed with permission of local Animal Health and Welfare Committees and in accordance with guidelines and regulations of the local Home Offices. Animal work should be carried out using country-specific guidelines and regulations.

### Method 1

Although the metatarsal bones of the foot develop in a similar manner to the long bones, it is only metatarsals two through five that develop true growth plates. Commonly, the metatarsal organ culture is established using postnatal (PN) mice at stages PN1–3. This helps delineate the mechanisms surrounding bone growth, as the primary ossification centre has completely developed in the metatarsal bones at this stage. It has, however, previously been recognised that PN bones have limited growth potential in culture.^31^ As such, metatarsal bones from embryonic (E) stages E17/18 are commonly used for this technique as the primary ossification centre has begun to form.^32^ More recently, earlier embryonic stages (E15) have been used, as these bones have yet to start ossifying and as such matrix mineralisation can be more precisely examined.^33^

### Specific materials

#### Preparation medium

PBS (phosphate-buffered saline) with 13% MEMα (Minimum Essential Media alpha) without ribonucleosides and 0.2% BSA (Fraction V); culture media: MEMα medium without ribonucleosides supplemented with 0.2% BSA (Fraction V), 5 μg ml^−1^ L-ascorbic acid phosphate, 1 mM β-glycerophosphate, 0.05 mg ml^−1^ gentamicin and 1.25 μg ml^−1^ fungizone; equipment: dissecting microscope, curved microscissors and fine forceps (for example, Dumont #5).

NOTE 1: Quality dissection equipment is required; in particular, the forceps and scissors should be kept sharp so as to ensure successful and easy dissection.

#### Animals

Pregnant dam at the desired stage or PN pups.

NOTE 2: When choosing the age of metatarsal bone culture, it is important to consider the outcomes required. For examination of promoters of matrix mineralisation, it is recommended that E15 is used. For assessment of endochondral bone growth over a long time period and inhibition of mineralisation, either E17/E18 or PN bones are recommended. As discussed above, PN bones do have a limited growth potential in culture and, as such, <PN3 is recommended if sufficient growth is required for data analysis.

### Embryonic and PN metatarsal dissection

**Figure 2:**
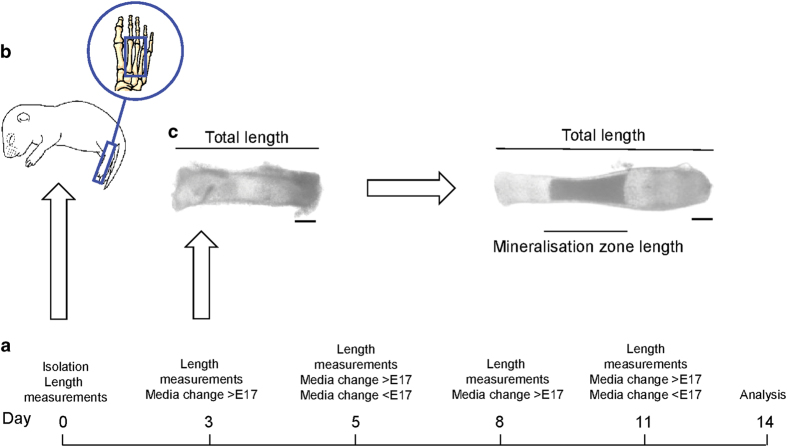
Timeline for the mouse metatarsal culture. On day 0, the middle three metatarsal bones from either embryonic (E) or postnatal mice are dissected (**a**) and cultured for up to 14 days. During this time, periodic measurements of the total length and the length of the mineralisation zone are made. Scale bar=200 μm. (**b**) Media changes are required every third day for metatarsal bones at embryonic stage E17 and above and postnatal metatarsal cultures (>E17). However, for metatarsal bones extracted at an embryonic stage earlier than this, e.g. E15 (<E17), it is not recommended to change the media for at least 5 days after dissection. The media can then be changed every third day. (**c**) Change in bone growth during explant culture.

1. Conduct all steps aseptically using sterile equipment and in a tissue culture hood to maintain a sterile environment.
2. If isolating embryonic metatarsals, cull the pregnant dam at the desired stage using cervical dislocation. If harvesting PN metatarsals, cull the PN pups by decapitation and go straight to step 5.
3. Fix the dam on her back by her legs and arms. Open the abdominal cavity with a single cut to expose the uterus.
4. Lift and cut out the uterine horns, placing them into a 10-cm culture dish. Remove the individual foetuses from their sacs and placenta.
5. Remove the hind limbs of the pups and place in preparation media until required.
6. Under a dissecting microscope, remove the skin from the hind limb by holding the upper hind limb with forceps and gently inserting the curved scissors and cutting down the back of the hind limb into the footpad. Then, separate the skin from the bones gently, as if pulling off a glove.NOTE 3: In the dissection of E15 metatarsals, skin removal by scissors is not required and access to the unmineralised metatarsals is best carried out by the careful use of fine forceps to remove overlying tissue. Because of the much smaller size and less rigid nature of E15 metatarsals, this is a much more delicate procedure than that in older E17/18 and PN pups.
7. Holding the tarsals in the foot with forceps, gently disrupt the connective tissue between the individual metatarsals by running the fine forceps in between them.
8. Remove the first and fifth metatarsals by pinching off with forceps and discard them.
9. Separate the phalangeal and tarsi bones from the remaining middle three metatarsals by careful pinching off by forceps (do not attempt to cut or use a scalpel; Figure 2a). Transfer into the preparation medium until all metatarsal bones are dissected.

NOTE 4: Although every effort should be made to remove the surrounding connective tissues, any remaining tissue will disappear after 24 h in culture.

### Metatarsal culture

1. Culture metatarsal bones individually in a well of a 24-well plate with 200 μl per well of culture medium.
2. Place in an incubator at 37 °C/5% CO_2_.
3. E17/E18 and PN metatarsal cultures may have the media changed every third day throughout the culture period. However, it is not recommended to change the media, at least for the first 5 days, when metatarsal bones at an embryonic age of less than E17/E18 cultures are used, as this prevents their matrix mineralisation capability (Figure 2b). After 5 days, the media on embryonic metatarsals <E17 can then be changed every third day.
4. Culture metatarsals bones for up to 2 weeks depending on the outcomes required.

### Analysis of endochondral bone growth and matrix mineralisation using metatarsal cultures

**Figure 3:**
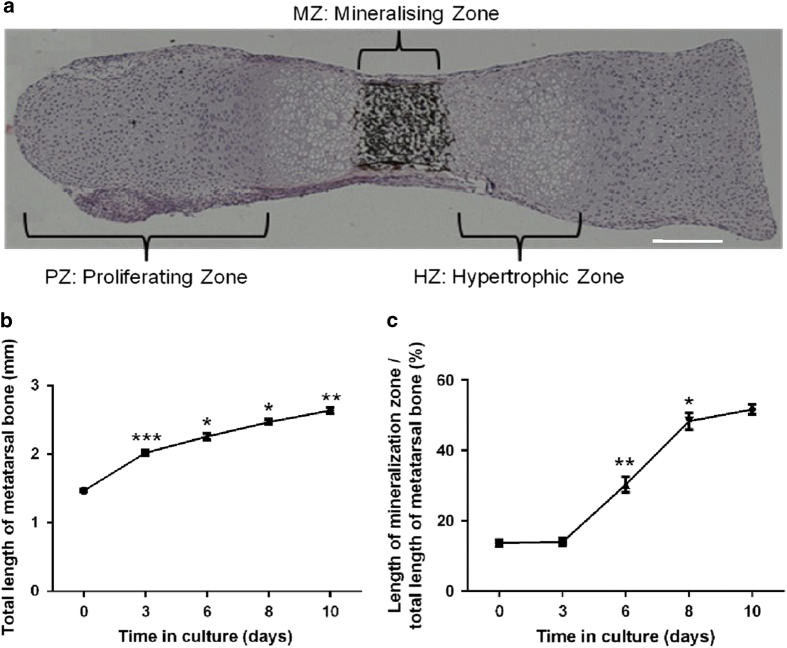
Mineralisation of metatarsal explants during culture. An E17 metatarsal bone was stained with Haemotoxylin and Eosin, and von Kossa stain after 7 days in culture. Clearly visible are the proliferative (PZ) and hypertrophic (HZ) zones of chondrocytes as defined by their well-characterised morphology, as well as the mineralisation zone (MZ) stained black by von Kossa staining. Scale bar=200 μm. (**a**) Total length increases of E18 metatarsal bones cultured for up to 10 days (n=6) (**b**). Increase in total mineralisation zone length as a percentage of the total length of the E18 metatarsal bones (*n*=6). Significance is in comparison with previous culture time point, *P<0.05; **P<0.01; ***P<0.001 *n*=6 metatarsal bones (**c**).

In culture, metatarsal bones will grow at a near physiological rates, and as such total bone length measurements indicative of longitudinal endochondral growth can be made over the culture period using a microscope with a camera attached and image analysis software^32^,^33^,^34^ (for example, ImageJ program; Figures 2c and 3a). Metatarsals may be cultured with [^3^H]-thymidine^35^ or BrdU^32^ in the final hours of culture and then examined by a liquid scintillation counter or processed to wax, respectively and reacted with an anti-BrdU antibody, to examine cell proliferation. Following culture, bones can be paraffin embedded and sections cut for standard haematoxylin and eosin (H&E) to examine the morphology and sizes of the resting, proliferative and hypertrophic zones of chondrocytes (Figure 3a).^33^

Longitudinal measurements of the central mineralisation zone can also be made to examine the rate of chondrocyte matrix mineralisation. This can be done by directly imaging and then using image analysis software, as described in Figures 2c and 3b and c. This is, however, a two-dimensional measurement; therefore, it is recommended that further analyses are performed so as to fully determine the mineralisation status of the metatarsal bones post culture, for example, using micro-computed tomography (microCT; see additional methods for details).

### Method 2

The *ex vivo* culture of femoral heads isolated from young mice is being predominantly used in cartilage research^36^ because of the developing femoral head consisting mainly of proliferating chondrocytes.^9^ However, when mice become around 3 weeks old, the chondrocytes undergo hypertrophy and are being slowly replaced, first by woven (around 6- to 9-week old) and then by trabecular bone (around 9- to 12-week old).^9^ Importantly, secondary ossification of the femoral head begins when animals reach adulthood.^37^ Thus, the equilibrium between markers associated with cartilage or bone will change during animal development. The state of tissue remodelling or response to specific stimuli can be measured directly by analysing the conditioned medium, extracting proteins from the explanted tissue, or by studying tissue histomorphology on fixed specimens. Here we describe a step-by-step technique for the isolation and culture of femoral heads from adult mice that allows for analysis of cartilage and bone markers.

### Specific materials

#### Culture medium

DMEM (Dulbecco's Modified Eagle Medium) with GlutaMAX (Waltham, MA, USA) and antibiotics (100 μg ml^−1^ streptomycin, 100 U ml^−1^ penicillin); PBS; 4% paraformaldehyde (PFA)/PBS solution, and 15% ethylenediaminetetraacetic acid disodium salt (EDTA)/0.5% PFA solution; equipment: blunt and fine scissors, fine and curved forceps, scalpel and sterile blades.

#### Animals

Balb/C mice at 11 weeks of age or C57BL/6 at 15 weeks of age.

NOTE 5: It is important to consider the genetic background of mice, as each strain may vary in endochondral and secondary ossification processes and overall cartilage matrix composition and bone structure.^38^

NOTE 6: It is recommended to use a minimum of 4 femoral heads (dissected from 4 mice) per experimental condition by placing each explant in a separate well of a 96-well plate. Each treatment group will consist of 4 biological replicates, which allows for sample matching and more robust statistical analysis, for example, using paired Student's *t*-test. If longer culture times or different analysis is required, then two femoral heads can be placed together in a well of a 48-well plate as described by Stanton *et al*.^36^

### Dissection of mice

1. Kill an appropriate number of mice by cervical dislocation.The remaining procedure must be performed in a sterile class II cabinet.
2. Spray mouse legs with 70% ethanol for disinfection.
3. Pull up the skin in the mid-abdomen region and cut it with blunt scissors to reveal muscles and internal body.
4. Peel off as much of the skin as possible to avoid contamination with fur.
5. Spray hind legs with 70% ethanol.
6. Using a scalpel blade, make incisions on both sides of the spine vertebrae. Cut the muscles surrounding the pelvic girdle using dissection scissors. Cut the pelvis in half and gently remove each leg together with the intact acetabulum. Make sure that the hip joint is intact and remains attached to the pelvic bone. Repeat the process for the second leg.

### Isolation of femoral heads

**Figure 4:**
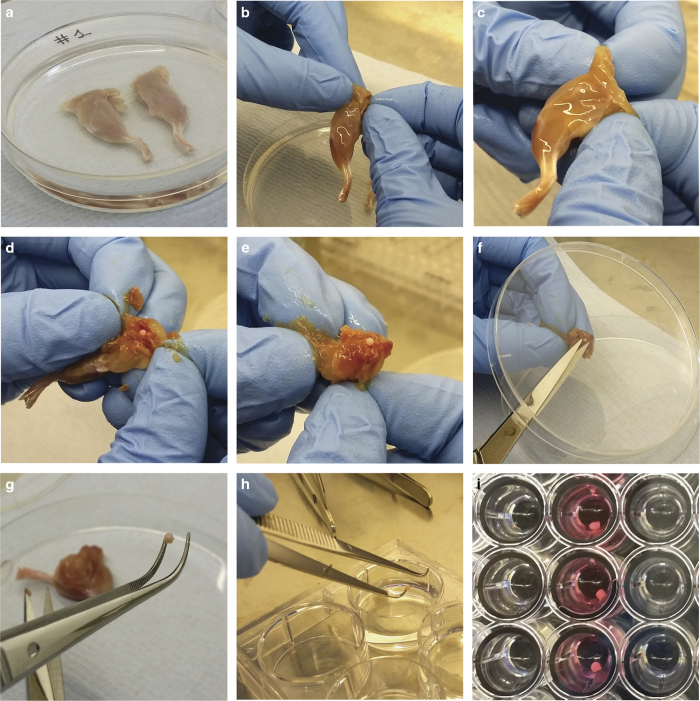
Isolation of mouse femoral heads. Balb/c mice (11-week old) were euthanised and legs were isolated according to steps 1–7. Both intact hip joints and legs were dissected and kept in PBS (**a**). Hip joints were disarticulated by breaking the pelvic bone in half and exposing the femoral head (**b**–**e**). The femoral neck was dissected with sharp scissors and washed in PBS before transferring each head into separate wells of a 96-well plate (**f**–**i**).

1. Transfer both legs into a 10-cm culture dish containing 20 ml sterile PBS solution (Figure 4a).
2. Pick up one of the legs using sterile forceps.
3. Grab both sides of pelvic bone with your fingers so that the acetabulum remains in the middle (Figure 4b).NOTE 7: Move your fingers as close to the joint capsule as possible, as this will help break the pelvic bone.
4. Using your fingers, break the pelvic bone in half to disarticulate the femoral head (Figure 4c).
5. Turn the leg over and gently pull each part of pelvic bone in opposite directions to expose the femoral head (Figures 4d and e).
6. Transfer the leg underneath the lid of the sterile 10-cm culture dish (to prevent the femoral head from drifting away) and cut off the femoral neck using sharp scissors (Figure 4f).
7. Lift the tissue with sterile forceps (Figure 4g) and place the explant into the well of a six-well plate containing 5 ml of sterile PBS (Figure 4h).
8. Disarticulate the remaining hip joint by repeating steps 1–7.
9. Place each of the heads into 200 μl of pre-warmed medium DMEM into a well of a 96-well plate (Figure 4i). Use only middle wells and add sterile PBS to the surrounding wells to prevent excessive evaporation.
10. Incubate the femoral heads at 37 °C and 5% CO_2_ for the time required in your experiment. Keep the explants for a maximum of 3 days without medium exchange.

NOTE 8: The femoral heads can be left in the culture for up to 10 days without significant loss in cell viability as mentioned in Madsen *et al*.^9^

**Figure 5:**
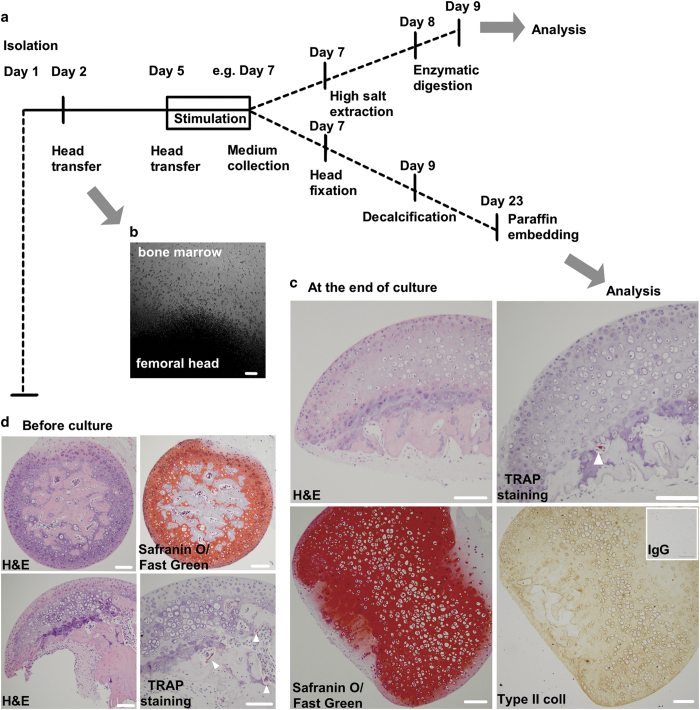
Analysis of cartilage and bone markers from cultured femoral head explants. In order to perform a specific stimulation, harvested femoral heads should be placed in culture (**a**). It is recommended to transfer the explants before stimulation in order to remove bone marrow contamination and to establish equal baseline culture conditions before treatment. Scale bar=100 μm. (**b**) Conditioned medium is collected at the end of stimulation. The remaining tissue is extracted using high salts such as guanidine hydrochloride or NaCl, which is followed by enzymatic digestion. Otherwise stimulated explants are fixed, decalcified and used for histology and immunohistochemistry. Femoral heads were isolated from C57BL/6 mice. Heads were extracted and fixed immediately, decalcified and processed into paraffin blocks. Tissue sections were stained with H&E, Safranin O/Fast Green or processed for TRAP activity or type II collagen expression in cartilage extracellular matrix. Scale bar=100 μm. (**c**) Femoral heads were isolated from Balb/C mice. Heads were extracted, cultured for 9 days and then fixed, decalcified and processed into paraffin blocks. (**d**) Tissue sections were stained as in **c.** Scale bar=100 μm.

NOTE 9: It is recommended to transfer the explants twice into separate wells of a 96-well plate, before starting the experiment, in order to remove bone marrow contamination. It also helps to establish equal baseline culture conditions before performing any stimulation on the explants (see Figure 5b).

In order to analyse the femoral head culture, the conditioned medium can be collected and the tissue fixed in PFA/PBS following decalcification in EDTA/PFA before processing and paraffin embedding.

### Conditioned medium collection

1. At the end of stimulation/treatment, aspirate 200 μl of medium from each well.
2. Centrifuge the medium for 10 min at 1500 *g* to remove any remaining cells. Transfer 180 μl of aspirate into a new collection tube.
3. Add 4.5 μl of 200 mM Tris/HCl stock (5 mM final concentration) into each sample with centrifuged medium in order to prevent pH changes during the freeze-thawing process. Store samples at −80 °C.

### Explant fixation

1. After collecting medium, wash femoral heads with sterile PBS.
2. Transfer heads into a fresh 96-well plate.
3. Fix heads with 4% PFA/PBS for 48 h at 4 °C and then decalcify tissue with 15% EDTA/0.5% PFA for 2 weeks at 4 °C. Each time add 300 μl of solution per head. Change the solution every 2–3 days.NOTE 10: Buffered formalin can be used as well to fix femoral heads, but for some applications PFA gives more flexibility with immunohistochemistry. Fix the explants with 10% buffered formalin for 48 h at 4 °C and decalcify tissue using neutral 12.5% EDTA/NaOH (pH 7) for 2 weeks at 4 °C.
4. Wash heads 3 × with 300 μl PBS (1 h per wash) before processing and embedding into paraffin blocks.
5. Embed femoral heads in the paraffin blocks and cut tissue into 3 μm sections using a microtome. Place one section on each slide.

NOTE 11: Try to embed the femoral head in the sagittal position, which helps orientate the sample and enables visualisation of cartilage and bone structure on the same tissue section.

### Analysis of cartilage and bone marker turnover using femoral heads cultures

Explant culture times and different approaches for comprehensive analysis are summarised in Figure 5. Briefly, changes in cartilage and bone markers during *ex vivo* explant cultures can be performed by (I) assessing the release of molecules into the conditioned medium, (II) analysing expression of molecules after extraction of the remaining tissue (III) or (III) by investigating the presence of specific tissue components and/or antigens on tissue sections. In the example shown here, the cartilage and bone structure was visualised by H&E stain (Figures 5c and d). The expression of proteoglycans and type II collagen within the cartilage was evaluated using Safranin O/Fast green and anti-type II collagen-specific antibodies, respectively. The osteoclast activity in trabecular bone was detected by TRAP (tartrate-resistant acid phosphatase) reaction.

The analysis of femoral heads can be accompanied by quantification of markers released into the conditioned medium, as shown by others. Examples of markers, which can be studied include type II collagen turnover (such as CTX-II or PIINP),^9^ proteoglycan depletion,^5^,^8^,^36^,^39^ non-collagenous proteins secretion,^39^ type I collagen fragments (CTX-I) release, hydroxyproline content or TRAP activity for bone.^9^

### Method 3

The neonatal calvaria is an active, not fully calcified bone tissue, routinely used to study resorption and bone formation *ex vivo*.^40^ In neonatal calvaria, bone modelling is favoured over bone remodelling and therefore might not always be the best model to study bone metabolism. However, culturing calvaria explants allow preserving cellular diversity and interactions within the bone microenvironment. Moreover, calvaria explants are excellent models to study the effect of cancer cells or their secretome on skeletal structure.^13^ Here we provide a method for mouse calvaria isolation, culture and characterisation that allows for the investigation of cancer cell-induced osteolysis.

### Specific materials

Isolation and culture medium: MEMα medium with 10% fetal bovine serum (FBS), 2 mM L-Glutamine, 100 U ml^−1^ penicillin and 100 μg ml^−1^ streptomycin; PBS, pH 7.2; equipment: scissors, tweezers and forceps (straight and curved fine tip), straight microscissors (12 mm cutting edge) and stainless steel mesh (can be obtained at the local hardware store, stainless steel no. 30 mesh is normally used).

**Figure 6:**
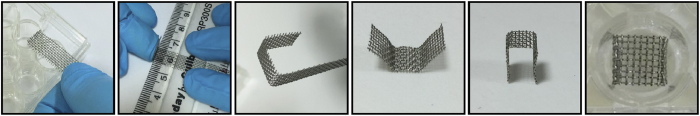
Preparation of stainless steel mesh for calvaria explant culture. Stainless steel meshes measuring 0.8 × 1 cm are cut from stainless steel sheets, and the two edges are bent to form a bridge that supports the calvaria. Meshes are then placed in the well of a 48-well plate.

NOTE 12: The entire mouse calvaria or the two halves can be cultured in 48-well plates on the stainless steel grid, prepared by cutting rectangular 0.8 × 1 cm pieces of stainless steel mesh from the sheet. Meshes are made by bending both ends with a ruler to make the bridge, as shown in Figure 6. The dimensions of the meshes should be adapted accordingly to the dimension of tissue culture plate. As described in Mohammad *et al*,^11^ four hemi-calvaria can be cultured at the same time on rectangular 1 × 1.5 cm meshes in a 12-well plate. After each use, wash the meshes with detergent and rinse them with 70% ethanol. The washed meshes are then rinsed with PBS to remove any residual detergent, air-dried and autoclaved.

#### Animals

C57BL/6 mice at 4–7 days old.

NOTE 13: Different strains of mice can be used for this assay with similar results. It is, however, important to compare treatments using a single pup calvaria divided into two halves due to the variability in osteogenic responses among litters and even among pups within the same litter.^40^

### Calvarial isolation

**Figure 7:**
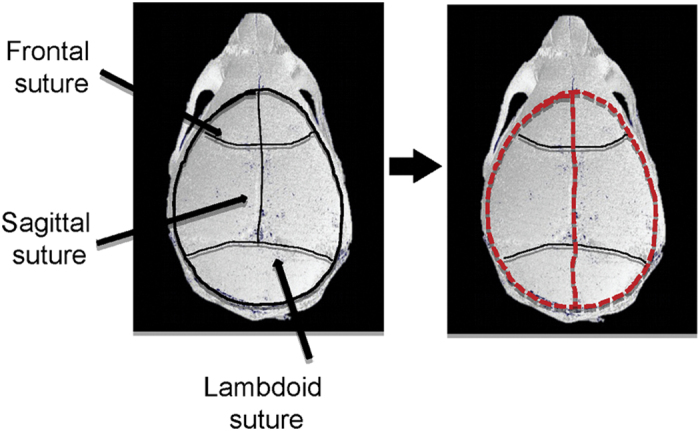
Anatomy of the mouse calvaria and its dissection. The red dotted line illustrates the isolation procedure.

1. Sterilise all tools and perform the isolation under sterile conditions.
2. Hold the pup with forceps and dip it in 95% ethanol for 2 s.
3. Decapitate the pups and transfer the heads into a 50 ml collection tube filled with 45 ml of sterile PBS. Keep the heads in PBS while dissecting one head at the time.
4. Using the curved forceps, firmly hold the head by placing the forceps on either side of the head.
5. Using the straight microscissors, remove most of the scalp. Expose the calvaria by incising both lateral sides of the calvaria and by moving the scissors from the eyes to the back of the skull. At the end, flip the excessive skin over the mouse forehead.
6. Identify the internal sutures of the calvaria as shown in Figure 7 and accurately cut the calvaria around the edges.
7. Gently remove the calvaria from the rest of the skull, remove the adherent connective tissue and wash the calvaria in PBS.
8. While holding the calvaria with straight fine tip forceps, cut it into two halves along the median sagittal suture and place each half in fresh medium.
9. Repeat the steps for all remaining calvaria.

### Calvarial culture

**Figure 8:**
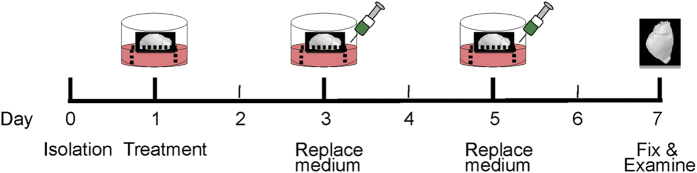
Timeline for the calvarial explant culture. On day 0, calvaria are isolated, cut into 2 halves and placed into separate wells of a 48-well plate containing 1 ml of complete medium. On day 1, the medium is replaced with a fresh medium either with or without cytokines, tested compounds or conditioned medium from cancer cell lines. On day 3 and 5, replace the medium with fresh medium either with or without cytokines, tested compounds or conditioned medium. On day 7, calvaria halves are removed, fixed and scanned by microCT for histological and histomorphometric examination.

1. Place one sterilised mesh into each well of a 48-well tissue culture plate.
2. Using the fine tip forceps, transfer half of the calvaria from the medium onto the top of the grid. Make sure that the concave or cranial side is facing down on the stainless steel mesh.
3. Add 1 ml of culture medium into each well.NOTE 14: Avoid flushing the calvaria with the medium. It is important that the calvaria half sits at the air–liquid interface in order to prevent the tissue from drying out or floating in the medium; 1 ml of medium is sufficient to cover calvaria when culture is performed in a 48-well tissue culture plate.NOTE 15: Use at least five calvaria halves from the same litter for each treatment group and an appropriate control.
4. Replace the medium 24 h later by carefully aspirating the medium with a syringe and adding 1 ml of medium containing the desired treatments. Change the medium every 48–72 h until the end of the experiment (Figure 8). The standard protocol is 7 days of treatment, but the duration can vary from 4 to 14 days.

### Calvaria fixation and paraffin embedding

1. At the end of the culture period, remove the calvaria halves from the mesh, transfer into a 1.5 ml collection tube and directly fix in 4% buffered formalin/saline (pH 7.4) for 24 h.NOTE 16: After fixation, rinse samples with PBS and store in 70% ethanol.
2. Rinse samples with PBS and decalcify in 14% EDTA pH 7.2 for 48 h.
3. Transfer samples into 70% ethanol before processing for paraffin embedding.NOTE 17: Following the dehydration procedure, place the samples in a freshly prepared paraffin-based infiltration solution for 4 h at 60 °C. Using forceps, transfer the calvaria into the molds filled with some wax. The sagittal suture should face down, toward the base of the mold, to ensure the correct orientation. Move the mold to a cold surface and make sure that the calvaria maintain the desired orientation while the paraffin wax hardens.
4. Cut 5 μm sections with a microtome, along the middle suture, and collect them every 20 μm interval on glass slides. Allow the slides to dry overnight before staining.

### Analysis of cancer-bone metastases using calvaria cultures

**Figure 9:**
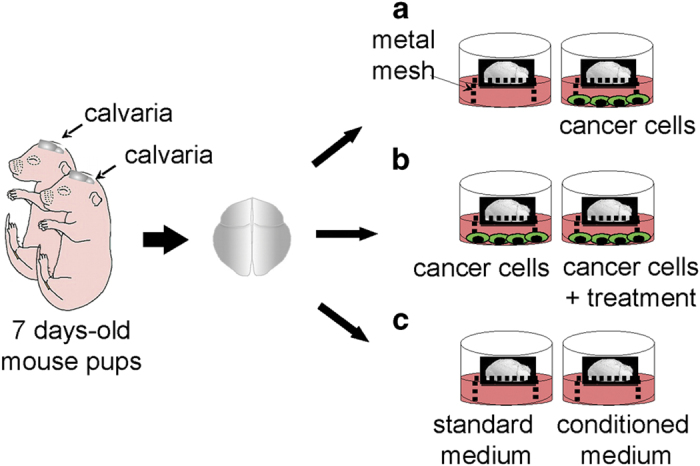
Model for cancer-induced osteolysis of the calvaria. Calvaria are divided into two halves. Each half is placed in the 48-well plates on the stainless steel meshes. Calvaria are grown in the absence or presence of cancer cells (**a**), in the presence of cancer cells with or without treatment (**b**), in standard medium or cancer cell-derived conditioned medium (**c**). Note: cancer cells should be seeded 24 h in advance. Medium is replaced every 48 h and the culture is terminated after 7 days.

In order to study the effect of cancer cells on bone cells *ex vivo*, calvaria are divided into two halves, and each half is placed on a stainless steel mesh in wells containing a medium with either no cells or cancer cells (Figure 9a). Cancer cells are seeded 24 h in advance to allow their adherence.

NOTE 18: Seeding density of the cancer cells is cell specific and has to be determined in advance.

NOTE 19: Recently, Curtin^41^ described a three-dimensional model in which a single calvaria is cultured in 2 ml of serum-free DMEM in the presence of 5 × 10^5^ floating cancer cells per tube or 25% of their conditioned medium; 150 μg ml^−1^ sodium ascorbate can be added to the culture medium in order to study the effect of cancer cells and exogenous factors on bone formation. Medium is refreshed every 48–72 h and the culture is terminated after 7 days.

NOTE 20: In addition, calvaria can be grown in the presence of a conditioned medium from cancer cells (20% v/v) or a medium containing either vehicle alone or exogenous factors to be tested (Figures 9b and c). Conditioned medium from cancer cells is obtained by culturing cancer cells in 6-well plates until they reach 80% confluence. Complete medium is then replaced with a serum-free medium. Cancer cells are cultured for an additional 16 h, and the conditioned medium obtained is removed and filtered through a 0.2 μm filter. Control standard medium is a serum-free medium incubated for 16 h at 37 °C and 5% CO_2_ in a six-well plate without cancer cells.

### Method 4

Previous work has demonstrated that rat mandibles can be extracted, sliced and kept viable in culture for at least 21 days.^16^ This method was later modified to include the use of green fluorescent protein (GFP) expressing DPSCs, enabling the visualisation of transplanted cells following injection into *ex vivo* mandible slice.^17^ Although much of the work on DPSCs has been completed using monolayer culture systems, there is a fundamental need to better understand how these cells behave in a three-dimensional system, providing a more accurate reflection of their *in vivo* characteristics.

### Specific materials

*DPSCs medium*. MEMα with nucleosides supplemented with 20% (v/v) FBS, 100 U ml^−1^ Penicillin, 100 μg ml^−1^ streptomycin sulphate and 100 μM L-ascorbic acid; PBS; collagenase-dispase (4 μg ml^−1^ in DPSCs medium); fibronectin (10 μg ml^−1^ solution diluted in PBS with 0.1 mM MgCl_2_ and 0.1 mM CaCl_2_); accutase; CyGel (Biostatus, Shepshed, UK); equipment: scissors, scalpel, tweezers, low-speed bone saw (IsoMet Buehler, Lake Bluff, IL, USA); 40 μm cell strainer, No 1.5 coverslips, blue agarose beads (BD Bioscience, Oxford, UK), nanofil syringe (World Precision Instruments, Sarasota, FL, USA).

*Animals*. 28-day-old Wistar rats and GFP rats (SDT(CAG-EGFP) CZ-004 Osb) produced as described elsewhere.^42^

### Preparation of mandible slice culture

**Figure 10:**
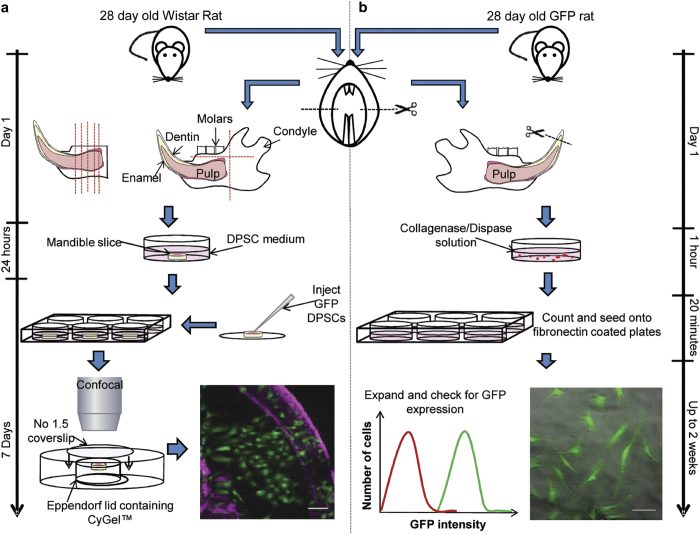
Mandible slice culture preparation and culture in the presence of DPSCs. (**a**) Images of injected GFP DPSCs after 7 days in culture (Green: GFP DPSCs, Magenta: reflected light from the mandible slice surface). Isolation and expansion of GFP DPSCs from a 28-day-old GFP rat. (**b**) GFP expression in cells can be observed using fluorescence microscopy or flow cytometry. Scale bar=100 μm.

Perform the isolation and culture of mandible slices as previously described in detail^16^ and follow these steps as an additional guidance:

1. Euthanise a 28-day-old Wistar rat and spray liberally with 70% ethanol.
2. Use scissors to make incisions on either side of the jaw (indicated by dotted lines in Figure 10) to increase exposure of the mandibles.
3. Use a scalpel to cut between the mandibles to loosen them.
4. Carefully dissect the soft tissue surrounding the mandible to remove it in one piece and submerge in the mandible in 70% ethanol for 10 s.
5. Remove any remaining soft tissue from both condyles as well as molars before slicing it into 2 mm transverse section using a bone saw as illustrated in Figure 10a.
6. Place one transverse section per well of a 24-well plate in 1 ml of DPSC media and stabilise overnight at 37 °C.

NOTE 1: Use a syringe and needle to inject a single blue agarose bead into the alveolar bone as previously shown^17^ so that the slice can be orientated at each imaging time point.

### Isolation of GFP DPSCs

All tissue culture procedures should be carried out in a sterile laminar flow tissue culture hood.

1. One day prior to DPSC isolation, prepare fibronectin-coated six-well plates with 1 ml per well of fibronectin solution. Seal the plates with parafilm and store at 4 °C overnight.
2. On day 2, remove mandibles from a euthanised 28-day-old GFP Wistar rat as described in steps 1–4 for mandible slice culture.NOTE 2: It is important to keep the mandibles intact, as pulp should only be removed from mandibles that remain in one piece.
3. Use the scalpel to cut the mandible to expose the pulp area and remove the pulp using tweezers.NOTE 3: This can often be achieved by cutting off the tip of the incisor to expose the pulp cavity, squeezing the mandible and removing the emerging pulp with tweezers.
4. Place the pulp in a 60 mm tissue culture dish containing 5 ml collagenase-dispase solution and mince finely using a scalpel.
5. Place in the incubator for 1 h.
6. Triturate the digested pulp using successively smaller pipette tips (1 ml, 200 μl and 10 μl) to obtain a single-cell suspension and finally pass through a cell strainer into a 50 ml collection tube to remove any remaining undigested tissue.
7. Centrifuge the cell solution at 400*g* for 5 min and re-suspend in 1 ml serum-free DPSC medium.
8. Count the cells using a haemocytometer and prepare a cell suspension of 1 × 10^4^cells per ml in the appropriate total volume.
9. Remove fibronectin solution from the six-well plates and replace with 1 ml of DPSC cell solution.
10. Incubate for 20 min at 37 °C.
11. Remove the medium and non-adherent cells, replace with DPSC medium and return the plate in the incubator.
12. Change the medium every 2–3 days until the cells reach 85–95% confluency.NOTE 4: It can take 15–18 days for cells to reach confluence.
13. Passage cells by removing the medium and washing once with PBS. Aspirate PBS and replace with 500 μl accutase and return the plate into the incubator for 5 min.
14. Once cells have detached, add 2 ml of DPSC medium, collect the cell suspension in a 15 ml collection tube and centrifuge at 400 *g* for 5 min to pellet the cells.
15. Aspirate the supernatant, re-suspend in 1 ml DPSC medium and count the cells. Re-seed at a density of 4 × 10^3^ cells per cm^2^.
16. Check for the presence of GFP using fluorescence microscopy.

### Analysis of DPSC behaviour in mandible slices

1. Dissociate GFP DPSCs at approximately 80–90% confluence using accutase as described above in steps 13–16 for GFP cell isolation.
2. Count cells using a haemocytometer and prepare a cell suspension of 2 × 10^6^cells per ml in the appropriate total volume.
3. Inject 1 μl of cell solution into the pulp of the mandible slice using a 35-gauge micro-needle and a nanofil syringe.
4. Return the mandible slice to the incubator until required for imaging, changing the media every 2–3 days.

The imaging of the mandible slice presents a challenge due to the presence of both soft and hard tissue. CyGel is optically clear and is used to reversibly mount the mandible slice, allowing collection of both fluorescence and reflected light images. These reflective light images provide reference points for imaging within the slice. This is especially useful for longitudinal studies. This method used has previously been described in detail.^17^

1. Remove the lid from a 1.5 ml collection tube and attach it to a 60 mm 10-cm dish with adhesive.NOTE 5: Ensure that the mandible slice is suspended with blue agarose beads facing up to allow the slice to be orientated and images collected with reference to the bead.
2. Cover the sample with a No 1.5 coverslip and place in the incubator for 3 min.NOTE 6: CyGel is a thermo-reversible hydrogel that gels rapidly at 37 °C, and therefore prepare the chamber with the gel using cooled pipette tips and keep the CyGel cool until required.
3. Collect GFP fluorescence using 488 nm excitation and 530/30 emission and reflection images at 488 nm using an upright confocal microscope.
4. Following imaging, rinse the chamber with 4 °C PBS to liquefy the CyGel and remove the mandible slice. Return the mandible slice to DPSC media in the 24-well plate.

NOTE 7: CyGel is biocompatible with live cells; therefore, cultures can be viably maintained following imaging.

### Method 5

The bovine trabecular explant model described below represents a system, in which osteocytes within their native environment can send signals to osteoblasts on the bone surface.^23^,^24^ Bone cores are thoroughly cleaned to remove bone marrow, disrupted nerves and vasculature, as studies have demonstrated an influence of these systems on bone modelling. This procedure has also been optimised to remove all surface cells from the trabecular bone explants and preserve only the osteocytes, which remain viable and embedded within the bone matrix. Then, a controlled number of primary osteoblasts are re-seeded onto the bone surface. Mechanical loading is subsequently applied by a custom loadable perfusion bioreactor to evaluate long-term histological and mechanical property changes within *ex vivo* grown bovine bone cores.

### Specific materials

#### Culture media

MEMα medium with 10% FBS and antibiotics (100 μg ml^−1^ streptomycin, 100 U ml^−1^ penicillin); PBS; 0.05 and 0.25% trypsin-EDTA; WD-40 (San Diego, CA, USA); LIVE/DEAD Viability/Cytotoxicity kit (Invitrogen, Waltham, MA, USA); equipment: diamond-tipped coring tool, 7 mm diameter (Starlite Industries, Rosemont, PA, USA); 60 ml sterile syringe and needles (18 and 30 gauge); Roccal-D Plus detergent (Zoetis); custom cell seeder, small magnetic stirrer; large pronged forceps, scalpel and blades; hand drill with locking bit and key; small vise; Interplak water jet (Conair, Stamford, CT, USA); IsoMet low-speed saw with high carbon (HC) wafering blade (Buehler, Lake Bluff, IL, USA); magnetic stir plate; mechanical testing device such as Bose Electroforce (Bose, Eden Prairie, MN, USA)

#### Animals

Bovine fetlock joints from 6-week-old calves can be obtained from a local slaughterhouse and shipped overnight on ice. The carpal-metacarpal joint contains the hoof, metacarpus and carpal joint and is usually cut at the radius and ulna. If the limb was cut higher, you may also see the elbow joint where the radius would connect with the humerus.

### Calf joint dissection

1. If the joint arrived with the skin, begin by gently removing the skin from the underlying muscle tissue with the scalpel. Examine the carpal joint and ensure that the joint capsule is intact. While handling the joint, be careful not to puncture the joint capsule to maintain sterility.NOTE 1: If the joint capsule has been punctured, isolate this joint from the others, as it may be more prone to infection.
2. Remove the hoof at the fetlock joint, where the metacarpus meets the hoof. To help determine the site of incision, lightly bend the joint and look for the space between the bones. There may already be an incision in the hoof from the killing process.
3. There are large tendons at the fetlock joint that need to be cut to remove the hoof. Also, trim these tendons along the shaft of the metacarpus, as they may cause the bone to slip once it is placed in the vise for drilling the bone cores. If you wish to cut off additional tissue at this point, you can remove some of the muscle surrounding the radius and ulna, but this is not necessary as this portion of the limb will not be used for the experiment.
4. Soak cut and skinned joints in a diluted antibacterial detergent for 30 min (use Roccal-D Plus or a similar product).
5. Transfer joints to 70% ethanol for another 30 min.

### Obtaining trabecular bone cores

**Figure 11:**
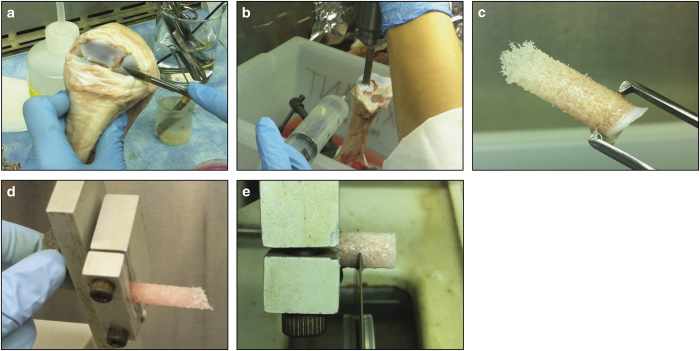
Bovine trabecular bone core isolation. (**a**) The carpal-metacarpal joint of a bovine calf fetlock. (**b**) Drilling bone cores through the medullary cavity of the metacarpus. (**c**) A trabecular bone sample. (**d**) Isomet low-speed saw assembly. (**e**) 7 mm trabecular bone explant cut from the bone core.

1. Transfer the joints to a sterile biosafety cabinet. Wipe the joint area with alcohol and hold the joint flexed. Cut open the carpal-metacarpal joint; the space between the metacarpus and the adjacent (first) carpal (Figure 11a). Cut off the radius and ulna.
2. The metacarpus should have a relatively flat articular surface. Using gauze soaked in 70% ethanol, wipe away the synovial fluid and clean the surface of the joint.
3. Wrap gauze on the bottom end of the carpal bone (opposite the end of the articular surface of interest). Put the end of the bone wrapped in gauze in the vise and tighten until the bone is stable.
4. Open a sterile syringe and fit it with a 18-gauge needle. Fill the syringe with PBS and rinse the surface of the bone.NOTE 2: Make sure that the exposed surface of the bone does not dry out at any point during the isolation procedure. In the following step, the bone should be constantly irrigated with PBS during drilling.
5. Put the coring tool into the drill and tighten the chuck with the key. Dip the coring tool in 70% ethanol and then rinse well in PBS. To drill, enter at an angle and lightly squeeze the trigger to drill at a slow speed (Figure 11b). The angle needs to be determined by looking at the overall shape of the bone; for example, if the midshaft is narrow and the articular surface is really wide, you must drill at a larger angle from the centre axis in order to avoid drilling through the cortical shell. Apply a light downward force on the drill but take care to lift up at times to allow PBS to enter the space between the drill bit and the bone. Stop drilling when the bone “gives” and hits the medullary cavity.NOTE 3: It is important to not drill too quickly, otherwise the bone around the edge of the bit will burn and a hard shell will be created.
6. Pull the coring tool out of the bone and remove the tool from the drill. Use a hex key to push the bone core out of the backside of the coring tool, pushing on the articular cartilage. Push the core into a 10-cm dish with 15 ml of pre-warmed, fully supplemented media. Repeat for as many bone cores as desired.

### Cutting bone cores

1. Sterilise the reservoir of the water jet with 70% ethanol, and then rise well with PBS.
2. Pick up the bone core with the pronged forceps (Figure 11c) and begin spraying PBS at the core to lightly clean the cores and remove some of the marrow.
3. Once all the cores are cleaned, set up the IsoMet. Set the speed between 2 and 3 to cut the bone at a slow speed. Fill the trough with enough PBS so that the blade is just skimming the surface of the PBS. Using sterile forceps, put a bone core into the holder, with the articular surface facing the IsoMet and the trabecular bone facing outwards (Figure 11d). Turn the blade on first, then slowly lower the specimen onto the blade. Trim the ‘rough' edge and remove the soft trabecular/marrow tissue.
4. Advance the micrometre to obtain a core that is 7 mm in height. Turn the blade on, then slowly lower the specimen onto the blade (Figure 11e). When the bone core is almost completely cut, hold the core with forceps so it does not fall. When the specimen is completely cut, place it in a 10-cm dish with fresh, pre-warmed, fully supplemented media.
5. Repeat until no more specimens can be cut from that core or until the specimen reaches the subchondral surface.

NOTE 4: The drill chuck, IsoMet components and vise should all be cleaned with water and WD-40 after use to remove residual salt from the PBS and prevent rust.

### Cleaning explants of bone marrow and surface cells

**Figure 12:**
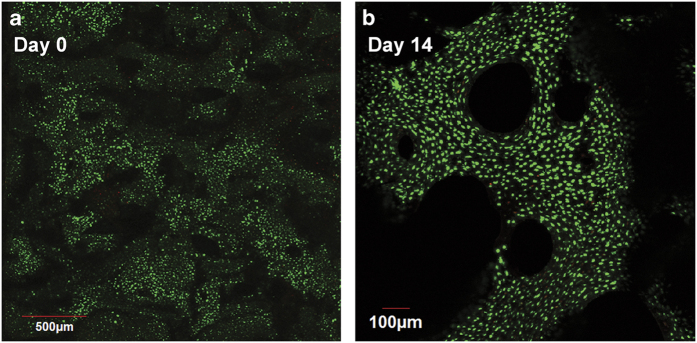
Cleaned trabecular bone explants. (**a**) A confocal image of a cleaned trabecular bone explant stained with a LIVE/DEAD cytotoxicity/viability kit. Note the absence of surface cells. (**b**) Cleaned explant after 14 days in culture. Live osteocytes remain in the interior and few cells re-populate the bone surface.

In the following steps, the individual bone explants are then thoroughly cleaned using PBS rinsing and trypsin treatments to remove bone marrow components, damaged vasculature, nerves and any surface cells. The only cells that remain after these steps are osteocytes embedded within the bone matrix (Figure 12a). The following procedure is robust so that, even after 2 weeks, very few surface cells remain to re-populate the bone surface (Figure 12b).

1. Once cut, thoroughly clean the bone cores with the PBS jet. The cores should be entirely white once the bone marrow is removed. Place the cleaned cores in a 12-well plate and cover with pre-warmed PBS.
2. To remove the surface cells, the cores must undergo a serial trypsin treatment. In each well, add ∼6 ml 0.25% trypsin-EDTA (enough to cover the cores) and incubate for 8 min. After incubation, remove the trypsin, neutralise any remaining trypsin by adding media to the wells and thoroughly rinse each core again with PBS. Repeat two more times so each core is treated three times with trypsin.
3. Rinse a final time with PBS. The final PBS rinse should be rather extensive to ensure that the detached cells on the bone surface are removed.
4. Put the cleaned cores in a new dish with pre-warmed, fully supplemented media.

### Seeding primary osteoblasts onto the explants

The bone cores may be used to obtain primary osteoblasts, but the “excess” bone cores pieces are also good for this procedure. Detailed methods for obtaining primary osteoblasts by explant outgrowth are described elsewhere.^43^

NOTE 6: The primary osteoblasts should be prepared in a previous harvest, as they take ∼3 weeks to expand.

**Figure 13:**
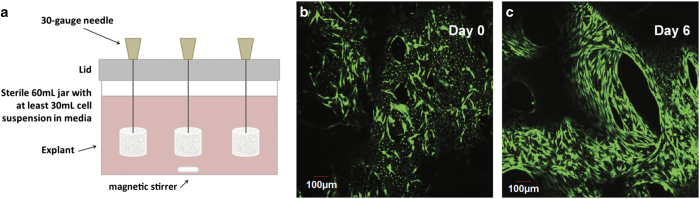
Seeding of primary osteoblasts. (**a**) Schematic of the custom cell seeder. (**b**) A confocal image of a seeded trabecular bone explant stained with a LIVE/DEAD cytotoxicity/viability kit. (**c**) Seeded explant after 6 days in culture.

The primary cells are used to seed a controlled number of cells back onto the bone cores using a custom cell seeder (Figure 13a). This achieves a uniform distribution of cells along the bone surface (Figure 13b). Osteoblasts proliferate and eventually cover the bone surface after a few days in culture (Figure 13c).

1. Trypsinise cells to be seeded onto bone cores and centrifuge to collect a cell pellet.
2. Count the cells using a haemocytometer to obtain a cell concentration. For osteoblast seeding onto 7 mm bone cores, the optimal concentration is 10^5^ cells per ml fully supplemented media. Prepare at least 30 ml of cell suspension. Pipet the cell suspension in the cell seeder jar with a magnetic stirrer.
3. Place the cell seeder lid on the benchtop so that the needles face upwards. Using forceps, skewer the bone cores onto the needles so that the flat side of the needles faces the lid and the cores are secure.NOTE 7: When attaching the bone cores to the needles of the cell seeder, try to push the needle through the trabecular pores rather than break through trabeculae.
4. Carefully place the lid on the cell seeder jar. Place the stir plate in a CO_2_ incubator at 37 °C and put the cell seeder on the stir plate. Stir slowly for 1 h.
5. Remove the lid from the jar, take the bone cores off the needles and transfer to a new culture dish with fully supplemented media.
6. Obtain the cell concentration of the remaining solution to estimate the seeding density per core, assuming uniform cell seeding for all cores.

### Transferring explant into loadable perfusion bioreactor

**Figure 14:**
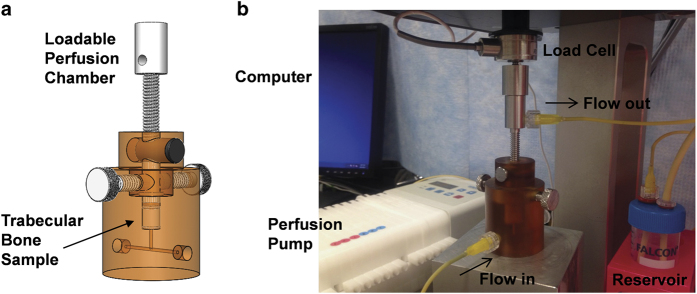
Loadable perfusion bioreactor. (**a**) Schematic representation of the bioreactor, indicating position of the trabecular bone sample. (**b**) Setup of the experimental system.

To sustain the viability of osteocytes in long-term culture, bone explants should be placed in a loadable perfusion bioreactor (Figure 14). In addition to traditional histological assessment, live osteocytes can be imaged by confocal microscopy using a fluorescent viability stain.

NOTE 7: In order to image the interior of the bone cores, they should be cut vertically in half using the Isomet saw prior to this procedure.

1. Prepare the LIVE/DEAD Cytotoxicity/Viability kit according to the manufacturer's instructions and use 2 μM calcein-AM and 4 μM EthD-1.
2. Remove the media from the bone cores and rinse once with PBS. Transfer the bone cores to sterile microcentrifuge tubes.
3. Add ∼1 ml of the working solution to each bone core and cover the tubes with foil to protect from light. Incubate the cores at room temperature for 45 min.
4. Image using a confocal microscope. Focus on the bottom of the bone core. You should see considerable numbers of dead cells here from the cutting procedure. Using the micrometre, focus at least 100 μm into the bone tissue to image and assess an undamaged region.

**Figure 15:**
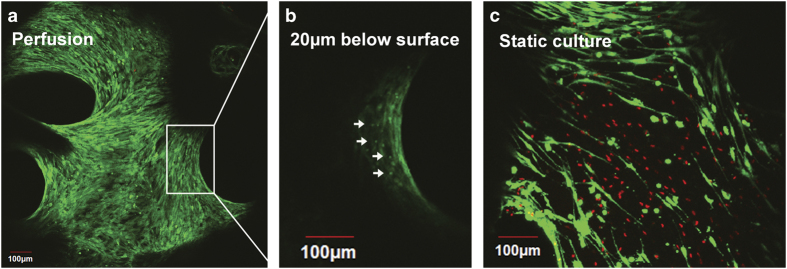
Perfusion system for maintaining osteocyte viability during long-term cultures. (**a**) Reconstructed confocal image of an explant perfused for 14 days in culture. (**b**) Inset of the confocal slice corresponding to the bone surface. Note: live osteocytes can be observed 20 μm below the bone surface. (**c**) Dead osteocytes (red staining) can be observed in the static cultures.

This method can be then used to evaluate the influence of perfusion on osteocyte viability and the general health of the cultures (Figures 15a-c). After 2 weeks in culture, live osteoblasts are confluent along the bone surface, and beneath this surface layer live osteocytes can be identified (Figure 15b). Static cultures, in comparison, show a considerable number of dead osteocytes (Figure 15c).

### Analysis of osteocyte viability and bone formation responses to mechanical loading

The perfusion bioreactor can be coupled to a mechanical testing device such as the Bose Electroforce. This setup can be used to apply dynamic, deformational loads to induce bone formation responses.

**Figure 16:**
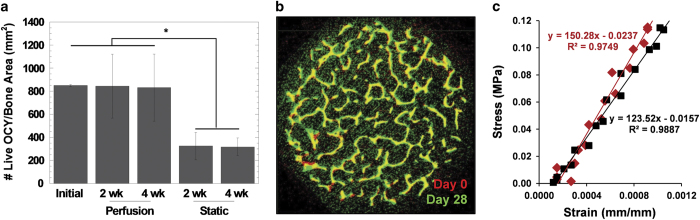
Assessment of osteocyte viability and bone responses to loading. (**a**) Histological assessment of osteocyte viability in explants cultured for 2 or 4 weeks (wk) in loadable perfusion bioreactors compared with static controls. (**b**) Registered microCT images from before (day 0) and after (day 28) the application of load. (**c**) Changes in elastic modulus determined by mechanical testing.

**Figure 17:**
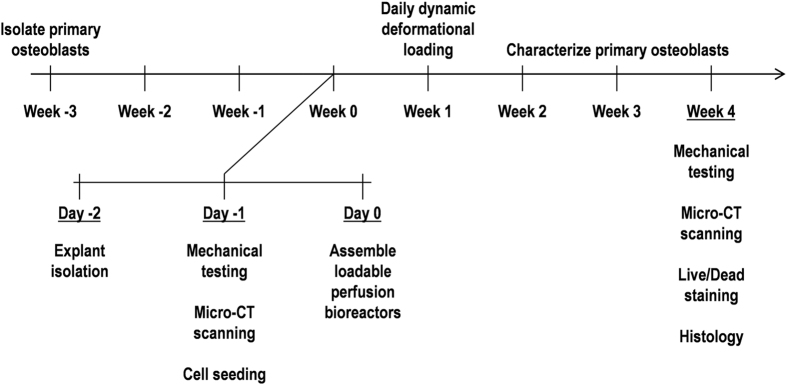
Experimental timeline for bovine explant culture and mechanical testing.

To complement staining in living explants, traditional histological techniques can be used to evaluate the bone. Explants can be fixed, embedded, sectioned and stained with traditional assays such as H&E. Comparing microCT scans from before and after the culture period using techniques such as image registration enables quantification of bone volume and microstructural changes (Figure 16a). Furthermore, mechanical testing of individual explants before and after the application of mechanical loading can be used to determine an effect of applied load on the apparent elastic modulus (Figure 16b). Combined, all listed techniques can be used to demonstrate long-term changes to short-term mechanical stimulation mediated by osteocytes (Figure 17).

### Additional methods to analyse *ex vivo* grown bone explants

#### Histological examination

Explants can be frozen, paraffin or plastic embedded. Bone explants are commonly decalcified before paraffin embedding. However, metatarsal bones do not need decalcifying for paraffin embedding and subsequent processing. Thus, histological methods such as von Kossa or alizarin red staining can be immediately adopted.^33^ Alkaline phosphatase (ALP) activity within the metatarsal bones can also be determined using a commercially bought assay for ALP.^33^ Similarly, calvarial bone can be embedded in plastic before von Kossa or TRAP reaction. In order to later detect TRAP activity on plastic, it is, however, important to perform the initial embedding and polymerisation steps at low temperature (4 °C), which partially preserves the enzymatic reactivity.^44^ Descriptions of staining for Toludine blue or Goldner's Trichrome, von Kossa or TRAP are in Supplementary Information.

#### Proteomic analysis

Conditioned medium from the mouse femoral head cultures can be monitored using Dimethylmethylene blue assay (detects sulphated glygosaminoglycans),^5^,^8^,^36^,^45^,^46^,^47^,^48^ western blotting^36^,^45^,^46^,^49^,^50^,^51^,^52^ or specific ELISAs.^9^

#### MicroCT

The quantification of bone parameters and visualisation of its microstructure can be performed by microCT.^53^,^54^ MicroCT analysis on the mouse metatarsals,^33^ mouse femoral heads^38^ and rat mandibular bone^55^ has been previously described. For the microCT analysis of the calvaria halves at the resolution of 5 μm gives an acceptable image quality when using Skyscan 1172 (Kontich, Belgium) or Scanco μCT35 (Brüttisellen, Switzerland) and following parameters: 60 kV and 150 μA, 0.5 mm aluminium filter, 0.6 degrees rotation angle. For microCT scanning of trabecular cores using Scanco VivaCT 40, resolution 10.5 μm and 300 ms integration time are recommended. This allows for high-resolution scans without affecting bone cell proliferation or differentiation by radiation.

#### Signalling mechanisms

Analysis of the signalling mechanisms can be successfully performed using the model cultures described in this protocol. The femoral head *ex vivo* model was effectively applied to study cell responses and the effects of cytokines (interleukin (IL)-1β,^5^,^45^,^47^,^49^,^50^,^56^,^57^ IL-1α,^48^ tumour necrosis factor-α^9^,^49^,^50^ or oncostatin M^9^), growth factors insulin-like growth factor 1 (IGF-1^9^,^49^), proteases^46^, alarmins^49^,^51^ or inhibitors^49^ on the cells and intact ECM. Similarly, metatarsal explants can be supplemented with different peptides, growth factors, recombinant proteins, antibodies or small-molecule inhibitors to examine their effects on bone development or on primary bone cells of the calvaria culture *ex vivo*.^58^,^59^ Finally, the inflammatory bone destruction can be studied using the mandibular slice model.^60^

#### Gene expression

Adenovirus techniques can be adopted to manipulate specific genes in metatarsal bones.^61^ RNA and protein can also be extracted from metatarsal bones when pooled together (∼4 bones per group) to provide sufficient quantities.^34^ Likewise, gene expression can be studied by isolating the RNA from the calvaria at the end of the culture period.^62^ In addition, defining the functional roles of individual genes can be achieved by modulating their expression by viral and non-viral methods on the calvaria organ culture or by manipulating primary osteoblasts prior to seeding onto trabecular bone cores.^63^,^64^

## Discussion

This protocol provides a detailed description of the most commonly used animal *ex vivo* bone culture models and gives examples of their application across different areas of bone research. Remarkably some of these explants can be isolated from genetically modified animals to greater our understanding of the role of specific genes in bone and cartilage biology. Although the main limitation of these models is a limited lifespan, the appropriate culture conditions indicated in this protocol and media supplementation enhance their use over a set period of time. Interestingly, *ex vivo* growth of metatarsal and femoral head explants are grown in the absence of serum, which eliminates the need of serum batch testing and improves reproducibility. However, for the long-term experiments, the serum supplementation and low-level perfusion driven by a peristaltic pump can be implemented to significantly extend culture periods and keep bone cells alive, as described here for trabecular bone explants.

The major advantage of these models is the ability to retain native bone cell communication and to study cellular responses in a physiological bone environment. For example, the metatarsal organ culture allows for direct examination of chondrocyte interactions during linear bone growth, which is not possible to observe using primary chondrocyte cultures. It also allows for the separation of systemic and local factors that affect bone development, therefore permitting the specific analysis of the local effects on the growth plate dynamics. Similarly, the trabecular bone explant model is unique in that it isolates the interactions between osteocytes and osteoblasts in a controlled manner to delineate mechanisms underlying load-induced bone formation responses.

Another advantage is that *ex vivo* culture models allow for the study of bone extracellular matrix remodelling. The model of mouse femoral head culture enables the study of matrix degeneration or *de novo* synthesis occurring simultaneously within the cartilage and bone. Likewise, the calvaria bone explant model is useful to enhance our understanding of mechanisms linked with bone resorption and formation. Finally, these models can be used to analyse novel interactions such as cancer cell-induced bone osteolysis or stem cell behaviour during bone repair. This indicates that there is a growing interest in the use of animal *ex vivo* bone culture models in several fields helping to increase our understanding about bone organotypic responses in health and disease.

## Recommended further reading

*Comprehensive review of various models to study endochondral ossification including the metatarsal organ culture system*: Andrade AC, Chrysis D, Audi L, Nilsson O. Methods to study cartilage and bone development. Endocr Dev 2011; 21: 52–66.

*Detailed characterisation of bone and catrtilage dynamic changes during mouse development*: Madsen SH, Goettrup aS, Thomsen G, Christensen ST, Schultz N, Henriksen K *et al*. Characterization of an *ex vivo* femoral head model assessed by markers of bone and cartilage turnover. Cartilage 2011; 2: 265–278.

*Comprehensive review of a three-dimentional model for the studies of cancer-bone metastasis ex vivo*: Curtin P, Youm H, Erdjan S. Three-dimensional cancer-bone metastasis model using *ex- vivo* co-cultures of live calvarial bones and cancer cells. Biomaterials 2012; 33: 1065–1078.

*Detailed protocol for establishing mandibular organotypic cultures*: Sloan AJ, Taylor SY, Smith EL. Organotypic mandibular cultures for the study of inflammatory bone pathology. In: *Replacing Animal Models*. John Wiley & Sons, Ltd, pp. 159–166.

*Application of loadable perfusion bioreactor to study biochemical and biomechanical changes of these bone cores over long-term cultures:* David V, Guignandon A, Martin A, Malaval L, Lafage-Proust M-H, Rattner A *et al*. Ex vivo bone formation in bovine trabecular bone cultured in a dynamic 3D bioreactor is enhanced by compressive mechanical strain. Tissue Eng Part A 2008; 14: 117–126.

## Supplementary Material

Supplementary Information
