# Supplementary material for: Models of ex vivo explant cultures: applications in bone research
Source: Bonekey Rep. 2016 Jun 29;5:818. doi: 10.1038/bonekey.2016.49 (PMC4926536; doi:10.1038/bonekey.2016.49)
Supplement: Supplementary Information [file bonekey201649-s1.doc]

*Supplementary information*

*NOTE 1:* The incubation times given below are optimised for calvarial bone and should be used as a guidance. We recommend optimising these methods if different bone tissue is used.

**Von Kossa/ H&E.** Stain the sections for 40seconds (in the dark) with 1.5 % aqueous silver nitrate solution (prepare fresh and filter before use) and rinse in deionized water three times. Develop the stain for 20-30seconds using 0.1% hydroquinone. Counterstain with haematoxylin and then with eosin. Mount with aqueous mounting medium and keep in the dark. Expected result: mineralised bone is stained black while the unmineralised bone is stained orange. Haematoxylin stains the osteoblasts.

**TRAP.** Sections should be reacted with a 0.45mm filtered solution made up of 150μl of Napthol-AS-BI-phosphate, 750μl veronal buffer, 900μl of acetate buffer, 900μl of acetate buffer with 100nM sodium tartrate, 120μl of pararosanilin and 120μl of 4% sodium nitrate. The incubation can vary between 30 minutes and 60 minutes but must be carefully monitored to avoid overstaining(64). Rinse in water and stain with haematoxylin for 45seconds. Rinse again and differentiate with acid alcohol for 8 seconds. Wash immediately and thoroughly in water and immerse in lithium carbonate for 30 seconds. Wash in water for 1 minute, rinse in 100% ethanol, immerse in xylene and then mount in DPX. Alternatively, commercially available kits such as leukocyte acid phosphatase staining kit are also widely used to visualize osteoclasts. Expected result: visualisation of osteoclasts stained in red. Haematoxylin can be used to counterstain.

**Toluidine Blue.** Sections are stained at acid pH 4.5 in a solution of 2% toluidine blue for 10-13 minutes. Expected result: mineralised bone is stained dark blue whereas the unmineralised bone is stained a light blue. Osteoblasts and osteocytes stain dark blue.

**Goldner’s Trichrome.** Prepare the following solutions; Solution A: 7.5mL Ponceau de xylidine (0.75% Ponceau, 0.25% acid fuchsin in deionized water + 1mL glacial acetic acid) + 2mL azophloxine (0.5% azophloxine in deionized water + 0.6mL glacial acetic acid) + 88mL 0.2% glacial acetic acid. Solution B Phosphotungstic acid /Orange G (3% phosphotungstic acid + 2% Orange G). Stain sections in Weigert’s haematoxylin for 20minutes, wash in water, then with 0.5% acid alcohol and wash again in water for 20minutes. Stain for 5minutes with Solution A. Rinse for 10seconds with 1% acetic acid and stain for 20minutes in Solution B. Rinse again for 10 seconds with 1% acetic acid and stain sections in 0.2% Light Green for 5minutes and rinse with water. Blot dry, rinse with 100% ethanol, immerse in xylene before mounting in DPX. Expected result: mineralised bone stains bright green and unmineralised bone stains red. Osteoblasts lining the surface of unmineralised bone and osteocytes are stained blue.
